# London Sanitation

**Published:** 1900-01-20

**Authors:** 


### The Hospital

A Journal of

The Medical Sciences and Hospital Administration.

Vol. XXVII.—No. 695.] Edited by Sir Henry Burdett, K.C.B., and Solomon C. Smith, M.D., M.R.C.P. [January 20, 1900.

### LONDON SANITATION.

The complication and multiplicity of the sanitary interests of the administrative county of London, and the amount of work which has to be performed by its officials with a view to the maintenance of the high standard of salubrity which obtains within its boundaries, are well set forth in the annual reports of the Medical Officer of Health for the county. The report for the year 1898, which has just been issued, forms, with its appendices, a volume of 193 folio pages, illustrated by 23 elaborate diagrams and two maps. It is divided into three principal parts, of which the first deals mainly with population statistics and with questions bearing upon their collection, or upon the interpretation which they should receive; the second, mainly with mortality and overcrowding; the third, mainly with the various forms and degrees of control which, in the interests of the public health, are exercised over the purveyors of various necessaries, or over the management of businesses or industries which are dangerous either to those engaged in them or to the residents in localities in which they are conducted. It would be impossible, within the limits of our space, even to enumerate the important questions which are discussed under one or other of these heads; and we can only select from the mine of wealth which the report, as a whole, places at our disposal. In any such selection perhaps the chief place should be given to a question to which Mr. Shirley Murphy was, we believe, the first to call attention, namely, the very manifest connection which exists between the work of London elementary schools and the diffusion of certain epidemic diseases, especially of scarlet fever and of diphtheria. About a week after the closure of the London Board schools for the summer vacation, which covers the last week of July and the first three weeks of August, the number of cases of both these diseases undergoes a sensible decline; and, about a week after the reassembling of the schools, the number undergoes a sensible increase. Making allowance for the period of incubation or of convalescence, it may be said that the notifications are at their highest point when the schools are in operation, and at their lowest point when the schools are closed. Taking the period covered by the years 1892-98, it appears that the notifications of scarlet fever at all ages have been fewer by 9·6 per cent. in August than in July, and more by 31·9 per cent. in September than in August, while within school ages the August decline was over 15 per cent., and the September increase rose to over 50 per cent. The diphtheria notifications in the same years for all ages were 16·2 per cent. fewer in August than in July, and 31·7 per cent. more in September than in August, while within school ages the decline was 30 and the increase 60 per cent. As a standard of comparison, Mr. Murphy takes enteric fever, as a malady which is notified, and which "for this purpose need not be thought of as communicable by school attendance." The August notifications in the same years were 25 per cent. in excess of those of July, and the September notifications were 46 per cent. in excess of those of August, so that the seasonal prevalence was clearly in no way diminished by school closure, nor dependent upon the same conditions as those which determined the prevalences of the other diseases concerned. It is satisfactory to find that the London School Board has become aware of the part which may be played by schools in the diffusion of epidemics, and that it has issued instructions to its teachers which go farther than the requirements of the Education Department. Teachers are instructed to exclude from attendance any child coming from a house in which infection is known to exist, and this instruction extends not only to the diseases subject to the notification provisions of the Public Health Act, but also to measles, chicken-pox, mumps, and whooping-cough. Moreover, teachers are required to give notice to the Medical Officer of Health of any children whom they exclude from school on account of infectious disease. Mr. Murphy points out, however, that, although these regulations are well designed for the purpose of dealing with recognised cases, they fail to provide against those which are too mild in their character to excite attention, and which only medical examination can detect. He expresses a strong opinion that such an examination should be systematically undertaken in every school which is suspected to be causing prevalence of disease, and that it should be undertaken either by the Medical Officer of Health for the district or by some medical man acting on his behalf. It would not be possible in a short time to examine numerous school children at their own homes, and the examination should, therefore, be conducted in the schools in which they are aggregated. It would, unquestionably, often serve to remove sources of danger, and might sometimes render it unnecessary for the sanitary authority to require the school as a whole to be closed. It is manifest that education, like many other things good in themselves, is capable of being a source of mischief or danger, and also that the mischief may be prevented, or the danger obviated, if adequate precautions for the purpose are enforced at the proper time.

